# Distribution of cervical intraepithelial neoplasia on the cervix in Chinese women: pooled analysis of 19 population based screening studies

**DOI:** 10.1186/s12885-015-1494-4

**Published:** 2015-06-27

**Authors:** Yu-qian Zhao, Irene J. Chang, Fang-hui Zhao, Shang-ying Hu, Jennifer S. Smith, Xun Zhang, Shu-min Li, Ping Bai, Wen-hua Zhang, You-lin Qiao

**Affiliations:** 1 Department of Cancer Epidemiology, Cancer Hospital Chinese Academy of Medical Sciences and Peking Union Medical College, 17 South Panjiayuan Lane, PO Box 2258, 100021 Beijing, China; 2 University of Miami Miller School of Medicine, 3303 Pinehurst Drive, Boynton Beach, FL USA; 3 Department of Epidemiology, Gillings School of Global Public Health, University of North Carolina, Chapel Hill, NC 27599 USA; 4 Department of Pathology, Cancer Hospital Chinese Academy of Medical Sciences and Peking Union Medical College, Beijing, 100021 China; 5 Department of Gynecology, Cancer Hospital Chinese Academy of Medical Sciences and Peking Union Medical College, Beijing, 100021 China

**Keywords:** Colposcopy, Cervical intraepithelial neoplasia, Lesion location, Biopsy, Cervical cancer

## Abstract

**Background:**

Controversy remains whether a pattern of cervical intraepithelial neoplasia exists on the cervix. Our study aims at determining if the prevalence of histologically proven lesions differs by cervical four-quadrant location or by 12 o'clock surface locations of diagnosis.

**Methods:**

We conducted a retrospective, histopathological study of 19 different population based cervical cancer screening studies from 1999 to 2010 by Cancer Hospital of Chinese Academy of Medical Sciences. The Institutional Review Board for human research subjects at CHCAMS approved all of the studies. During the colposcopy procedure, participant received either 4-quadrant biopsy or directed biopsy with/without endocervical curettage. Data of all samples were stratified by the methods of sampling. Kruskal-Wallis test was used to determine overall distribution of normal/CIN1, CIN2 and CIN3+ on the cervix.

**Results:**

In total, 53,088 cervical samples were included in distribution analysis. 66.9 % samples were obtained by random biopsy, 16.1 % were by directed biopsy, and 17.0 % were by endocervical curettage. 95.9%of the biopsied samples were diagnosed as normal/CIN1, 2.0 % were CIN2, and 2.1 % were CIN3 + . CIN2 and CIN3+ were most often found in quadrants 2 and 3 (χ_KW_^2^ = 46.6540, p < 0.0001) and at the 4- and 7-o'clock positions by directed biopsy (OR_CIN2_ = 2.572, 1.689, OR_CIN3+_ = 3.481, 1.678, respectively), and at the 5-, 6-, 7-, 9- and 12-o’clock positions by random biopsy. CIN3+ was least often found at the 11-o’clock position by directed biopsy (OR = 0.608).

**Conclusions:**

Our results suggest a predisposition of specific locations on the cervix to CIN occurrence. Quadrants 2 and 3, especially the 4- and 7-o’clock positions should be preferentially targeted during biopsy. The decision for random biopsy should be reconsidered in future studies.

**Electronic supplementary material:**

The online version of this article (doi:10.1186/s12885-015-1494-4) contains supplementary material, which is available to authorized users.

## Background

Persistent infection with high risk human papillomavirus (hr-HPV) has been established as the major etiological factor for cervical intraepithelial neoplasia (CIN) [1–3]. Early detection of precursor lesions is imperative because without treatment, all grades of CIN may progress to invasive cervical cancer, although CIN 1 lesions progress less frequently [4, 5]. Carcinogenesis occurs within the transformation zone of the cervix, where primary screening methods such as the Papanicolaou (Pap) smear detect early cytological abnormalities [4, 6]. Definitive diagnosis of CIN is obtained through colposcopy with biopsy and histopathology [7–10].

Colposcopy with directed biopsy is the current gold standard for diagnosis of pre-invasive cervical cancer, with sensitivity up to 84.8 % for high-grade squamous intraepithelial lesions or worse (HSIL+) [11]. Despite its high accuracy and concordance with histology, colposcopy technique remains largely operator-dependent with no standardized guidelines [12–14]. To address the practitioner-dependent limitations of colposcopically directed biopsy, colposcopists are recommended to obtain additional random biopsies from distinct locations, and to perform endocervical curettage (ECC) in women with ambiguous pap smears or women over 45 years old with suspected high-grade lesions [15–17].

Controversy exists in literature on whether there is a topographical pattern of CIN on the cervix that could be targeted by colposcopy [18–24]. The cervix is often identified by clockwise, using the o’clock position with the 12 o’clock and the 6 o’clock position being located at the midpoint of the anterior and posterior lip of the cervix, the 3 o’clock and 9 o’clock position located at the midpoint of the right and left side, respectively. Some researchers reported a predilection of histologically confirmed CIN loci for the anterior and posterior cervical os [18–21]. He et al. suggests that CIN lesions are not randomly distributed, but concentrated in the 12-, 8-, and 7-o’clock sites on the cervix [18]. Allard et al and Heatley M reported a predilection for the locations on anterior and posterior lips of the cervix [19, 20]. Richart claimed CIN occurs more frequently on the anterior lip of the cervix than on the posterior [21]. However, Yang HP et al have not found preferential sites on the cervix for CIN3 [22]. Besides, there are also some studies report heterogeneity in CIN occurrence across the cervix, but claiming the evidence maybe confounded by some factors, such as a tendency of the anterior and posterior lips to look more acetowhite, the inherent imprecision of colposcopy and operator bias for anterior-posterior cervical sampling due to mechanical ease [23, 24]. Clinicians were recommend to take multiple random biopsies during colposcopy in all cervical quadrants even without visible lesions to avoid missing CIN invisible to the naked eye [15, 16], a possible existing predilection distribution of CINs on the cervix may help the clinicians to make decisions while performing random biopsy. Since controversy still remains, our study aims to determine if the prevalence of histologically proven CIN lesions differs by cervical 4-quadrant location or by 12-o’clock location of diagnosis on the cervix. These findings may help in the development of colposcopy guidelines.

## Method

### Population

We conducted a retrospective, pooled data analysis of 19 different population based screening studies conducted by the Cancer Hospital, Chinese Academy of Medical Sciences (CHCAMS) in Beijing, China. We determined the distribution of CIN 2+ lesions among 38,633 women participating in studies from 1999 to 2010 listed in Additional file 1 a (i.e, Shanxi Province Cervical Cancer Screening Study(SPOCCS) I (1999), SPOCCS II (2001-2002), SPOCCS III-1-5 (2006-2007), Screening Technologies to Advance Rapid Testing(START) 2003, 2004, 2005, 2006, 2007, Screening Technologies to Advance Rapid Testing—Utility and Program Planning (START-UP) 2010, cooperative screening studies with International Agency for Research on Cancer(IARC) I, II and III, FastHPV trial (2007), Prevalence survey (2008), and Hybrid Capture (HC) 2 trial (2008)). The Institutional Review Board for human research subjects at CHCAMS approved all these studies prior to commencing. Written informed consent was obtained from all women. Study procedures and methodology have been described previously [25, 26].

Participants who were biopsied in all studies were between 19 to 65 years old, not pregnant, and had no history of pelvic surgery or irradiation. In colposcopy, the surface of the cervix divided by perpendicular lines drawn from 12- to 6- o’clock and from 3- to 9-o’clock. The four cervical quadrants are labeled clockwise, with quadrant 1 from 12 to 3 o’clock, quadrant 2 from 3 to 6 o’clock, quadrant 3 from 6 to 9 o’clock, and quadrant 4 from 9 to 12 o’clock. Screened women included in our analysis had at least one positive result on various cervical cancer screening tests (Additional file 1), except for women in the SPOCCS I trial which all participants underwent 4-quadrant biopsy and ECC regardless of their screening results and in START-UP study that 10 % of all primary screening negative women underwent colposcopy and 4-quadrant random biopsy and ECC. After being referred to colposcopy, according to the proposals (SPOCCS II, SPOCCS III, START 2003-2007), participants received colposcopically directed biopsy in any abnormal-appearing area and random biopsy in other negative quadrants at the squamocolumnar junction around 2-, 4-, 8-, and 10-o’clock so that participants in these studies referred to colposcopy had a minimum of 4 quadrants biopsies. In other studies (Prevalence study, HC2 trial, FastHPV trial and IARC 1-3), participants received directed biopsy at the positive colpscopy quadrant only or 4-quadrant biopsy were performed at the squamocolumnar junction if the colposcopy diagnosis were negative. ECC was subsequently performed according to study protocols. The indications for colposcopically directed biopsies were the same across the studies that any abnormal-appearing areas should be targeted, including suspicious HPV infection or low-grade lesions. The quadrants and/or o’clock location were required to be recorded by the operators. Only participants with complete biopsy records and pathological diagnoses were included. Samples with incomplete data, unsatisfactory biopsies, and biopsies with ambiguous diagnoses or non-specific labeling of location of origin (e.g., “close to 6 o’clock”, “between 2 and 3 o’clock”) were excluded. Cases with only quadrant but no o’clock data were included in the 4-quadrant analysis and excluded from the 12 o’clock location analysis. In studies with international collaborators, final diagnosis was based on the international pathologist’s read. In domestic studies, the final diagnosis was established by simple majority consensus among readings by three separate pathologists.

### Statistical analysis

Data of all samples were stratified into three groups based on method of colposcopic sampling – random biopsy, directed biopsy, or ECC, and analyzed using SAS9.2 software. Kruskal-Wallis test was used to determine overall distribution of normal/CIN1, CIN2 and CIN3+ on the cervix with statistical significance set at p < 0.05. Chi-square test was used to compare the difference of rates. Occurrence of cervical lesions was grouped by quadrants, then by 12 o’clock location. Differences in CIN distribution by quadrants and by o’clock location were analyzed using the Kruskal-Wallis test at the level of adjusted α’. The adjusted α_1_ for quadrant location was 0.0083 and α_2_ for o’clock location was 0.00075 respectively. Adjusted α values were calculated by the Bonferroni test (α’ = α/ [k*(k-1)/2], α = 0.05)). The 10 o’clock location, which had the relatively lower frequency of CIN occurrence, was used as the reference point of comparison for CIN occurrence in other o’clock locations.

## Results

In total, 38,633 women participated in the 19 screening studies. Of these 38,633 women, 12,656 were referred to colposcopy with biopsy and/or ECC. Participants with quadrants biopsies and/or ECC and a pathological diagnosis were included. Among the 12,656 women, 199 of them were excluded since biopsied only on polyps or missing data; 9001 women received four-quadrant biopsies and ECC; 1089 women received 4-quadrant biopsies without ECC; 283 women received one to three quadrants biopsies with ECC; 2013 women received one to three quadrants biopsy without ECC and 71 women had ECC only.542 women were diagnosed as CIN2, 484 CIN3 and 64 cervical cancer cases.

The sociodemographic data of participants received biopsy are shown in Table 1. Mean age was 41.5 with an average of 3 pregnancies, 2.3 live births, and an average of 1.5 lifetime sexual partners. Of the total 53,592 histopathology samples obtained, 382 samples were diagnosed as unsatisfactory or others. 122 samples lost information of biopsied type, among them, 4 CIN3 or worse (CIN3+), 6 CIN2 and 112 CIN1/Normal. 53,088 samples were included in distribution analysis. 95.9 % (50,912/53,088) of biopsied specimens were diagnosed as normal/CIN1, 2.0 % (1074/53,088) were CIN2, and 2.1 % (1102/53,088) were CIN3+. CIN2 or worse (CIN2+) lesions constituted 4.1 % (2176/53,088) of the total cases. 66.9 % (35,508/53,206) samples were obtained by random biopsy, 16.1 % (8538/53,088) were by directed biopsy, and 17.0 % (9042/53,206) were by ECC. Of the 44,046 samples obtained by quadrants biopsy, 2.2 % (973/44,046) were found to be CIN2 and 2.1 % (927/44,046) were found to be CIN3+. The positive rate of CIN2+ lesions by directed biopsy (14.1 %, 1201/8538) or random biopsy (2.0 %, 699/35,508) showed statistical significance (*χ*^2^ = 2440.635, p < 0.0001).

**Table 1:** Demographics of 12,656 biopsied participants

	Mean ± SD	Median (Range)
Age in years	41.5 ± 7.2	41 (19-65)
Age at menarche in years	15.7 ± 1.9	16 (10-26)
Sexual history		
Age at sexual debut in years	20.9 ± 2.3	21 (13-37)
Number of pregnancies	3.0 ± 1.3	(0-16)
Number of live births	2.3 ± 1.0	(0-14)
Number of sexual partners	1.5 ± 1.2	(0-40)
	Number (n)	Percentage (%)
Marital status		
Single	34	0.30 %
Married	12081	96.10 %
Widowed	235	1.90 %
Divorced	77	0.60 %
Education level		
<Primary school	1262	10.00 %
Primary school	4055	32.20 %
Middle school	6088	48.40 %
High school or above	1027	8.20 %
Current contraceptiveuse		
Yes	11510	91.50 %
Contraceptive method		
Oral contraceptive pill	269	2.30 %
Condom	473	4.10 %
IUD	3476	30.20 %
Sterilization	8913	77.40 %
Smoking history		
Never smoked	12061	95.90 %
Quit smoking	40	0.30 %
Current smoker	271	2.20 %

*SD* standard deviation; *IUD* intrauterine device

Of the 9042 samples obtained by ECC, 1.1 % of them (101/9042) were found to be CIN2 and 1.9 % (175/9042) were found to be CIN3+. The distribution difference of CIN2+ lesions by quadrants biopsy and ECC is statistical significant (4.3 % vs. 3.1 %, OR = 1.4318, *χ*^2^ = 30.3592, p < 0.001).

The distribution frequency of CINs by cervical location of all women, grouped by method of biopsy is summarized in Table 2.

**Table 2:** Distribution frequency of normal/CIN1, CIN2, and CIN3 + lesions by method of biopsy, grouped by cervical quadrant location

Quadrants		Diagnosis													
		Normal/CIN1				CIN2				CIN3+				Total	
		n	n/Nr(%)	95%CI		n	n/Nr(%)	95%CI		n	n/Nr(%)	95%CI		Nr	Nr/N(%)
*Directed biopsy*															
	Q1	1968	88.1	86.7	89.4	129	5.8	4.9	6.8	136	6.1	5.2	7.1	2233	4.2
	Q2	1583	83.2	81.5	84.8	167	8.8	7.6	10.1	153	8.0	6.9	9.3	1903	3.6
	Q3	1548	82.8	81.1	84.5	146	7.8	6.7	9.1	175	9.4	8.1	10.8	1869	3.5
	Q4	2238	88.4	87.1	90.0	126	5.0	4.2	5.9	169	6.7	5.7	7.7	2533	4.8
P < 0.0001															
*Random biopsy*															
	Q1	8698	98.2	97.9	98.5	87	1.0	0.8	1.2	72	0.8	0.6	1.0	8857	16.7
	Q2	8873	97.9	97.6	98.2	106	1.2	1.0	1.4	80	0.9	0.7	1.1	9059	17.1
	Q3	8717	97.8	97.5	98.1	118	1.3	1.1	1.6	77	0.9	0.7	1.1	8912	16.8
	Q4	8521	98.2	97.9	98.4	94	1.1	0.9	1.3	65	0.7	0.6	0.9	8680	16.4
*P* = 0.1911															
ECC															
		8766	96.9	96.6	97.3	101	1.1	0.9	1.4	175	1.9	1.7	2.2	9042	17.0
Total		50912	95.9	95.7	96.1	1074	2.0	1.9	2.1	1102	2.1	2.0	2.2	53088	100.0

*CIN* cervical intraepithelial neoplasia; *Q* quadrant; *ECC* endocervical curettage; *CI* confidence interval

Overall, CIN2+ lesions were significantly more frequently found in the posterior cervix (second and third quadrants, n = 1022) than in the anterior cervix (first and fourth quadrants, n = 878, *χ*^2^ = 15.556, p < 0.0001). When the cervix was divided in half on a sagittal plane, there was no significant difference in CIN2+ occurrence between the left (third and fourth quadrants, n = 970) and the right sides (first and second quadrants, n = 930, *χ*^2^ = 0.994, *p* = 0.319). By directed biopsy, CIN2 and CIN3+ lesions were significantly more likely to be found in the second and third quadrants than in the first and fourth quadrants (χ_KW_^2^ = 46.6540, p < 0.0001). CIN2 lesions obtained by directed biopsy were significantly more likely to be found in the second and third quadrants (n = 313) than in the first and fourth quadrants (n = 255), (χ_KW_^2^ = 35.3607, p < 0.0001). CIN3+ lesions were also significantly more frequently found in the second or the third quadrant (n_2_ = 153, n_3_ = 175) than in the first or fourth quadrant (n_1_ = 136, n_4_ = 169), (χ_KW_^2^ = 22.4373, p < 0.0001). No significant differences in quadrant distribution were found for CIN2 and CIN3+ lesions obtained by random biopsy (χ_KW_^2^ = 4.7494, *p* = 0.1911).

**Fig. 1:**
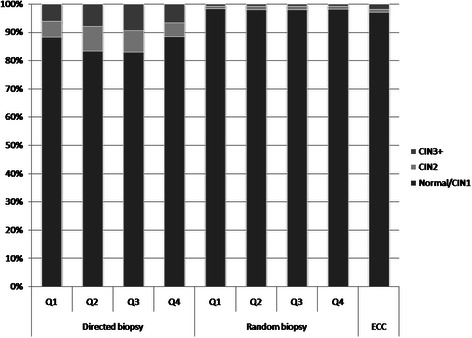
Frequency of normal/CIN1, CIN2, and CIN3+ by cervical quadrants, group by method of biopsy

Figure 1 shows the distribution frequency of CIN lesions by cervical quadrant location and grouped by method of biopsy of all the cervical samples.

**Fig. 2:**
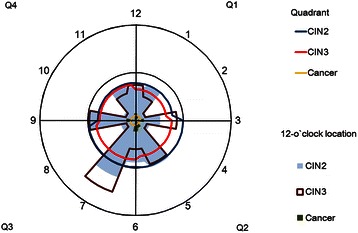
Topographical distribution of CIN2+ lesions by quadrant and 12-o’clock cervical location

Of the 53,088 samples included, the information of 12 o’clock location was not recorded for 11,594 samples, 107 samples without definite location information, so that 41,387 samples were included in clock location analysis. The distribution frequency of CIN by 12 o’clock location and grouped by method of biopsy is presented in Table 3. In both random and directed biopsy, there was a statistically significant pattern of CIN occurrence on the cervix (χ_KWd_^2^ = 54.3880, χ_KWr_^2^ = 73.1819, p < 0.0001). By directed biopsy, CIN2+ lesions were most likely to occur at the 4- (odds ratio, OR = 2.572, 95 % Confidence interval, 95 % CI: 1.900, 3.481) and 7- (OR = 1.689, 95 % CI: 1.211, 2.355) o’clock positions. The CIN3+ lesions were most likely to occur at 4- and 7-o’clock positions as well, the ORs are 2.959 (95 % CI: 2.026, 4.323) and 1.678 (95 % CI: 1.095, 2.572) respectively. By random biopsy, CIN2+ lesions were more likely to occur at the 5- (OR = 4.793, 95 % CI: 2.462, 9.330), 6- (OR = 3.841, 95 % CI: 1.530, 9.644), 7- (OR = 4.185, 95 % CI: 2.156, 8.121), 9- (OR = 3.657, 95 % CI: 1.125, 11.893), and 12-(OR = 3.697, 95 % CI: 1.593, 8.583) o’clock positions. CIN3+ lesions were more likely to occur at the 3- (OR = 6.033, 95 % CI: 1.431, 25.431), 5- (OR = 4.744, 95 % CI: 1.695, 13.277), 7- (OR = 5.178, 95 % CI: 2.046, 13.106) and 12-(OR = 4.575, 95 % CI: 1.408, 14.861) o’clock positions. A visual representation of the topographical distribution and severity of CIN on the cervix is shown in Fig. 2.

**Table 3:** Distribution frequency of normal/CIN1, CIN2, and CIN3 + lesions by method of biopsy, grouped by 12 o’clock cervical location

O’clock location	Diagnosis													
	Normal/CIN1		CIN2		CIN3+		Total		OR_CIN2+_	95 % CI		OR_CIN3+_	95 % CI	
	n	n/Nr(%)	n	n/Nr(%)	n	n/Nr(%)	Nr	Nr/N(%)						
*Directed biopsy*														
1	728	91.8	35	4.4	30	3.8	793	13.1	0.775	0.547	1.098	0.624	0.387	1.006
2	391	86.3	26	5.7	36	7.9	453	7.5	1.376	0.961	1.971	1.394	0.880	2.209
3	168	91.3	6	3.3	10	5.4	184	3.0	0.827	0.470	1.456	0.901	0.444	1.831
4	486	83.6	49	8.4	95	7.9	581	9.6	2.572	1.900	3.481	2.959	2.026	4.323
5	436	88.4	26	5.2	31	6.3	493	8.1	1.135	0.788	1.635	1.076	0.668	1.735
6	319	90.9	15	4.2	17	4.8	351	5.8	0.871	0.564	1.345	0.807	0.045	1.437
7	442	83.7	37	7.0	49	9.3	528	8.7	1.689	1.211	2.355	1.678	1.095	2.572
8	553	88.5	41	6.6	31	5.0	625	10.3	1.130	0.802	1.592	0.849	0.528	1.365
9	138	87.9	7	4.5	12	7.6	157	2.6	1.195	0.699	2.042	1.317	0.677	2.562
10	651	89.7	32	4.4	43	5.9	726	12.0	1	/	/	1	/	/
11	672	92.1	31	4.2	27	3.7	730	12.0	0.749	0.523	1.073	0.608	0.372	0.996
12	405	91.6	16	3.6	21	4.8	442	7.3	0.793	0.525	1.198	0.785	0.459	1.342
Total	5389	88.9	321	5.3	353	5.8	6063	100.0						
*Random biopsy*														
1	276	96.8	6	2.1	3	1.1	285	0.8	1.829	0.923	3.623	1.508	0.470	4.841
2	8321	98.3	80	0.9	67	0.8	8468	24.0	0.991	0.786	1.248	1.117	0.786	1.588
3	46	95.8	0	0.0	2	4.2	48	0.1	2.438	0.586	10.139	6.033	1.431	25.431
4	8646	98.1	94	1.1	74	0.8	8814	25.0	1.090	0.871	1.363	1.188	0.843	1.674
5	117	92.1	6	4.7	4	3.2	127	0.4	4.793	2.462	9.330	4.744	1.695	13.277
6	73	93.6	4	5.1	1	1.3	78	0.2	3.841	1.530	9.644	1.901	0.260	13.904
7	134	93.1	5	3.5	5	3.5	144	0.4	4.185	2.156	8.121	5.178	2.046	13.106
8	8488	97.9	108	1.2	70	0.8	8666	24.5	1.176	0.943	1.467	1.144	0.808	1.620
9	46	93.9	3	6.1	0	0.0	49	0.1	3.657	1.125	11.893	0	0	0
10	8187	98.2	87	1.0	59	0.7	8333	23.6	1	/	/	1	/	/
11	208	96.7	4	1.9	3	1.4	215	0.6	1.887	0.873	4.079	2.001	0.622	6.436
12	91	93.8	3	3.1	3	3.1	97	0.3	3.697	1.593	8.583	4.575	1.408	14.861
Total	34633	98.0	400	1.1	291	0.8	35324	100.0						

*CIN* cervical intraepithelial neoplasia; *OR* odds ratios; *CI* confidence intervals

## Discussion

The goal of colposcopy is to identify suspected high-grade lesions on the cervix and to rule out subclinical (or preclinical) invasive cancer. Ambiguity occurs in defining appropriate colposcopy practices and biopsy site selection, which leads to inaccurate diagnosis and treatment. Our study demonstrates significant distribution frequency of CIN2+ lesions on the cervix by quadrants, which may help colposcopists target specific regions on the cervix to obtain additional biopsies. CIN2 and CIN3+ lesions obtained by directed biopsy were more commonly found on the posterior two quadrants (quadrants 2 and 3). Previous studies have demonstrated increased CIN2+ diagnoses at the posterior cervix compared to the anterior [18]. Pretorius et al. also found a slightly increased prevalence of CIN2+ in the posterior cervix, which they attributed to verification bias [24]. Since specificity of colposcopy was not assessed in our present study, we are unable to draw conclusions about whether verification bias was present. The distribution frequency of CIN lesions in our study is not likely due to colposcopist preference for oversampling the anterior and posterior cervix due to mechanical ease [19, 22, 27], as approximately equal numbers of biopsies were taken from each quadrant, the opportunity to detect CIN2+ lesions in these quadrants should be equal. It is possible that with the greatly increased sample size in our study detected a true clinical difference in CIN2+ prevalence by cervical 4-quadrant location. Percentage of CIN2+ diagnoses was also significantly higher in quadrants 2 and 3 compared to quadrants 1 and 4, implying that independently of presence and grade of disease, location plays a role in lesion severity.

There was also significant frequency of CIN distribution by 12-o’clock location, further supporting that the specific points on the cervix may be predisposed to CIN growth. By directed biopsy, CIN2+ lesions were most likely to occur at the 4- and 7-o’clock positions, and least likely at the 11-o’clock position. This finding is consistent with He et al’s study, which found the most severe lesions at the 7- and 8-o’clock locations. While the 12-o’clock location was found to be the most common for CIN2+ lesions in both our studies, we found 11-o’clock instead of 2-o’clock as the least common location.

Although the results of directed biopsies taken from 12 o’clock cervical locations show non-random distribution of the lesions is reliable, we also noticed that no statistical significance was found by random biopsy in quadrants. This may be due to the increased diagnostic accuracy of directed biopsy for CIN2+ in larger, visible lesions [27] and only colposcopy invisible lesions would be found by random biopsy, causing the low detection rate of CIN2+ lesions by biopsy targeting normal-appearing areas [28], which in our study is 2.0 %. It is possible that since most random biopsy at normal-appearing areas performed at 2-, 4-, 8-, 10-o’clock, possible invisible lesions on the perpendicular midline of the cervix might be missed, which is the finding by directed biopsy in our study and also other researchers [18–21]. This may be an explanation to the low detection rate of CIN2+ lesion by random biopsy in our screening studies as well. By random biopsy, CIN2+ lesions were more likely to occur at the 5-, 6-, 7-, 9- and 12-o’clock positions rather than 2-, 4-, 8-, 10-o’clock. Considering this and the findings of directed biopsy, the decision on the positions for random biopsy should be reconsidered in future studies.

Strengths of this study are the large sample size, broad age range of participants, detailed labeling of biopsy location, rigorous methodology, and high level of diagnostic quality control based on three separate pathologist readings. Our aggregate results on the location and histopathological diagnosis of 53,088 cervical samples represent the most comprehensive analysis of CIN distribution frequency to date. Regardless of method of biopsy, there was an increased frequency of CIN2+ lesions on the posterior midline cervix. Possible etiology of the predilection of CIN for the anterior and posterior cervix may be twofold. First, mechanical trauma to the anterior and posterior cervix during intercourse, combined with decreased blood flow and pooling of fluids and sloughed squamous epithelium in the anatomical recesses may lead to lower viral clearance in the anterior and posterior fornices [23, 29]. Secondly, squamous transformation of the anterior and posterior lips of the cervix occurs later in embryological development than the lateral sides, allowing more time for malignant potential [23]. The squamocolumnar junction is formed by mesenchymal induction caudally, leaving some residual Wolffian duct segments within the endocervical stroma. This epithelial-mesenchymal transition process has been implicated in cervical carcinogenesis, which could explain CIN predominance in the posterior cervix [30, 31].

Weaknesses of this study are the retrospective design and non-uniform number of biopsies conducted at each of the 12 o’clock points on the cervix due to the inherent imprecision in colposcopy. However, clinicians in our study obtained comparable number of biopsies by quadrant. Women in our study were older and multiparous, limiting the generalizability of our findings to younger, low-risk populations. Future prospective studies on cervical conization samples may reveal the true distribution of CIN lesions. Randomized prospective studies comparing the diagnostic outcomes of women with additional biopsies taken from sites with greater CIN frequency may confirm our findings and evaluate if targeting certain sites, such as the perpendicular midline of the cervix for normal-appearing areas increases the detection rate of CIN lesions.

## Conclusions

The distribution pattern of CIN2+ lesions identified in our study has important implications for future screening and clinical management of precancerous cervical lesions. In the event of diffuse or equivocal changes in the cervix, Quadrants 2 and 3, especially the 4- and 7-o’clock positions should be preferentially targeted during biopsy as this may increase diagnostic accuracy. The decision on the position for random biopsy should be reconsidered in future studies.

## Additional file

Additional file 1:
**Summary of studies conducted from 1999 to 2010.**

## Abbreviations

CIN: Cervical Intraepithelial Neoplasia
CHCAMS: Cancer Hospital, Chinese Academy of Medical Sciences
CI: Confidence Interval
ECC: Endocervical curettage
HC2: Hybrid Capture 2
hr-HPV: High risk human papillomavirus
HSIL: High-grade Squamous Intraepithelial Lesions
IARC: International Agency for Research on Cancer
LSIL: Low-grade Squamous Intraepithelial Lesions
OR: Odds Ratio
Pap smear: Papanicolaou smear
SPOCCS: Shanxi Province Cervical Cancer Screening Study
START: Screening Technologies to Advance Rapid Testing
START-UP: Screening Technologies to Advance Rapid Testing—Utility and Program Planning
