# Supplementary material for: Distribution of cervical intraepithelial neoplasia on the cervix in Chinese women: pooled analysis of 19 population based screening studies
Source: BMC Cancer. 2015 Jun 27;15:485. doi: 10.1186/s12885-015-1494-4 (PMC4485364; doi:10.1186/s12885-015-1494-4)
Supplement: Additional file 1: — Summary of studies conducted from 1999 to 2010. [file 12885_2015_1494_MOESM1_ESM.pdf]

Appendix 1: Summary of studies conducted from 1999-2010

Study	Study year; location	Number screened	Age	Screening tests	Follow-up procedure	Histology or cytology location and review	Number biopsied
SPOCCS I	1999; Xiangyuan County, Shanxi Province	1997	35–45	HC2 (self, physician), fluorescence test, LBC, VIA, colposcopy	All women received four-quadrant biopsies and ECC under colposcopy	CICAMS, blinded international review	1997
SPOCCS II	2001–02; Xiangyuan and Yangcheng County, Shanxi Province	8497	35–50	HC2 (self, physician), LBC, VIA, AFB	positive VIA, self-test or physician-test for high-risk HPV, or an abnormal AFB, or a positive Pap test (ASC-US or worse):4-quadrant biopsies and ECC	CICAMS	3253
SPOCCS III-(1)	2006; Xiangyuan County, Shanxi Province	884	16–54	HC2 (self, physician), LBC, VIA	Positive VIA or positive self-HC2 (colposcopy and directed biopsy, ECC if necessary); positive physician-collected HC2 or ASC-H or worse on LBC (colposcopy and 4-quadrant biopsies, ECC if necessary)	CICAMS; blinded international review	185
SPOCCS III-(2)	2006; Beijing	795	16–54	HC2, LBC, VIA	Positive VIA (colposcopy and directed biopsy, ECC if necessary); positive physician-collected HC2 and ASC-US on LBC or ASC-H or worse (colposcopy and 4-quadrant biopsies, ECC if necessary)	Peking University People's Hospital, blinded international and CICAMS review	69
SPOCCS III-(3)	2006; Xinmi, Henan Province	879	16–54	HC2 (self, physician), LBC, VIA	Same as SPOCCS III-(1)	CICAMS, blinded international review (only histology)	164
SPOCCS III-(4)	2006; Yutian County, Xinjiang Uygur Autonomous Region	883	16–54	HC2 (self, physician), LBC, VIA	Same as SPOCCS III-(1)	CICAMS, blinded international review (histology); People's Hospital of Xinjiang Uygur Autonomous Region, blinded CICAMS review (cytology)	138
SPOCCS III-(5)	2007; Shanghai city	774	16–54	HC2, LBC, VIA	Positive VIA (colposcopy and directed biopsy, ECC if necessary); positive physician-collected HC2 or ASC-H or worse (colposcopy and four-quadrant biopsies, ECC if necessary)	Shanghai, blinded international and CICAMS review (for cytology, blinded CICAMS review only)	43
START 2003	2003; Xiangyuan County, Shanxi	2005	30–49	HC2, LBC, VIA	Positive VIA, HC2 positive, or ASC-H or worse on LBC (colposcopy and four-quadrant biopsies and	CICAMS, blinded international review	523

	Province				ECC)		
START 2004	2004; Xiushui County, Jiangxi Province	2499	30–49	HC2, LBC, VIA, VILI, colposcopy	Positive VIA, VILI, or colposcopy (four-quadrant biopsies and ECC); negative VIA or VILI but HC2 positive, or ASC-H or greater on LBC (repeat colposcopy and four-quadrant biopsies and ECC)	Jiangxi, blinded international and CICAMS review	537
START 2005	2005; Wudu County, Gansu Province	2053	30–49	HC2, LBC, VIA, VILI	Positive VIA or VILI (colposcopy and directed biopsy, and ECC if necessary); negative VIA or VILI but HC2 positive, ASC-H or worse on LBC (colposcopy and 4-quadrant biopsies and ECC)	Gansu Cancer Hospital, blinded international and CICAMS review (histology); CICAMS, blinded international review (cytology)	381
START 2006	2006; Qinxian County, Shanxi Province	2500	30–49	HC2, LBC, VIA, VILI, colposcopy	Same as START 2005	CICAMS; blinded international review	559
START 2007	2007; Xiangyuan and Wuxiang County, Shanxi Province	2530	30–54	HC2, careHPV, LBC, VIA, colposcopy	Positive VIA or colposcopy (directed biopsy, and ECC if necessary); negative VIA but HC2 or careHPV positive or ASC-H or worse on LBC (repeat colposcopy and four-quadrant biopsies and ECC if necessary)	CICAMS, blinded international review	691
START-UP	2010;Yangcheng County, Shanxi Province; Xinmi City, Henan Province; Tonggu County, Jiangxi Province	7543	25-65	HC2, careHPV, E6 test, VIA, colposcopy	Positive HC2, careHPV or E6 (positive colposcopy with directed biopsies, and ECC if necessary. Negative colposcopy with random biopsy and ECC); HC2, careHPV and E6 all negative(10% of the negative women undergo 4-quadrants random biopsy and ECC)	CICAMS	3197
IARC-(1)	2005; Shenzhen City, Guangdong Province	1137	15–59	HC2, LBC, VIA, colposcopy	Positive colposcopy (directed biopsy, and ECC if necessary); negative colposcopy, but HC2 positive and ASC-US, or LSIL or worse on LBC (repeat colposcopy with directed biopsies, and ECC if necessary)	Shenzhen, blinded CICAMS review	122
IARC-(2)	2004; Yangcheng County, Shanxi Province	745	15–59	HC2, fluorescence test, LBC, VIA, VILI, colposcopy	Positive colposcopy or fluorescence test (directed biopsy and ECC if necessary); negative colposcopy, but HC2 positive and ASC-US, or LSIL or worse on LBC (follow-up 1 year later)	Yangcheng County Cancer Hospital, blinded international and CICAMS review (for histology, blinded CICAMS review only)	306

IARC-(3)	2005;Shenyang City, Liaoning Province	719	15-59	fluorescence test, LBC, VIA, VILI, colposcopy	Positive VIA, VILI or fluorescence test (directed biopsy and ECC if necessary); negative colposcopy, but ASC-US, LSIL or worse on LBC (repeat colposcopy with random or directed biopsies, and ECC if necessary)	Liaoning Cancer Hospital, CICAMS review	243
*Fast*HPVtrial	2007; Qinxian County, Shanxi Province	818	30–50	HC2, careHPV, LBC, VIA, VILI	Either VIA or VILI or careHPV was positive (colposcopy and directed biopsy, and ECC if necessary); VIA and VILI and careHPV were negative, or colposcopy was negative, but HC2 positive or LSIL or worse on cytology (four-quadrant biopsies, and ECC if necessary)	CICAMS	63
Prevalence survey	2008; Binhai and JintanCounty, Xuzhou city, Jiangsu Province	316	18–25	HC2, LBC, VIA	Positive VIA (colposcopy and directed biopsy, and ECC if necessary); negative VIA but ASC-H or worse on LBC (colposcopy and four-quadrant biopsies, and ECC if necessary); negative VIA but HC2 positive and ASC-US or better on LBC (colposcopy and directed biopsy, and ECC if necessary)	CICAMS	40
HC2 trial	2008; XiangyuanCounty, Shanxi Province	1059	30–59	HC2, LBC, VIA, VILI	Positive VIA or VILI: directed biopsy, and ECC if necessary; negative VIA or VILI but HC2 positive and ASC-US, or ASC-H or worse on LBC (colposcopy and four-quadrant biopsies, and ECC if necessary); either HC2 positive or ASC-US on LBC (directed biopsy, and ECC if necessary)	CICAMS	145
Total		38633					12656

SPOCCS=Shanxi Province Cervical Cancer Screening Study. HC2=Hybrid Capture 2. LBC=liquid-based cytology. VIA=visual inspection with acetic acid. ECC=endocervical curettage. CICAMS=Cancer Institute andHospital of the Chinese Academy of Medical Sciences. AFB=Ampersand's fluorescentbiomolecular markers. ASC-US=atypical squamous cells of undetermined significance. ASC-H=atypical squamous cells cannotexclude high-grade squamous intraepithelial lesion. VILI=visual inspection with Lugol's iodine. START=Screening Technologies to Advance Rapid Testing. START-UP=Screening Technologies to Advance Rapid Testing—Utility and Program Planning
